# An evaluation of an influenza vaccination campaign targeting pregnant women in 27 clinics in two provinces of South Africa, 2015 – 2018

**DOI:** 10.1186/s12913-021-06962-8

**Published:** 2021-09-09

**Authors:** Kate Bishop, Meredith McMorrow, Susan Meiring, Sibongile Walaza, Liza Rossi, Sarona Mhlanga, Stefano Tempia, Azwifarwi Mathunjwa, Jackie Kleynhans, Grace D. Appiah, Johanna M. McAnerney, Heather J. Zar, Cheryl Cohen

**Affiliations:** 1 Centre for Respiratory Diseases and Meningitis, National Institute for Communicable Diseases (NICD), a division of the National Health Laboratory Service (NHLS), Johannesburg, South Africa; 2 Division of Public Health Services and Response, National Institute for Communicable Diseases (NICD), a division of the National Health Laboratory Service (NHLS), Johannesburg, South Africa; 3 Influenza Division, Centers for Disease Control and Prevention, Atlanta, GA USA; 4 Influenza Program, Centers for Disease Control and Prevention, Pretoria, South Africa; 5 DST/NRF Vaccine Preventable Diseases/Respiratory and Meningeal Pathogens Research Unit (RMPRU), Johannesburg, South Africa; 6 School of Public Health, Faculty of Health Sciences, University of the Witwatersrand, Johannesburg, South Africa; 7 Division of Global Migration and Quarantine, Centers for Disease Control and Prevention, Atlanta, GA USA; 8 Department of Paediatrics and Child Health, Red Cross War Memorial Children’s Hospital, and SA-MRC Unit on Child & Adolescent Health, University of Cape Town, Cape Town, South Africa

**Keywords:** Influenza vaccines, Pregnant women, South Africa, Vaccination

## Abstract

**Introduction:**

Despite prioritization, routine antenatal influenza vaccine coverage is < 16% in South Africa. We aimed to describe maternal influenza vaccine coverage in 27 antenatal clinics (ANCs) in Gauteng and Western Cape (WC) Provinces, where in collaboration with the Department of Health (DoH), we augmented the annual influenza vaccination programme among pregnant women.

**Methods:**

From 2015 through 2018, 40,230 additional doses of influenza vaccine were added to the available stock and administered as part of routine antenatal care. Educational talks were given daily and data were collected on women attending ANCs. We compared characteristics of vaccinated and unvaccinated women using multivariable logistic regression.

**Results:**

We screened 62,979 pregnant women during the period when Southern Hemisphere influenza vaccines were available (27,068 in Gauteng and 35,911 in WC). Vaccine coverage at the targeted clinics was 78.7% (49,355/62682), although pregnant women in WC were more likely to be vaccinated compared to those in the Gauteng (Odds ratio (OR) =3.7 *p* < 0.001). Women aged 25—29 and > 35 years were less likely to be vaccinated than women aged 18—24 years (OR = 0.9 *p* = 0.053; OR = 0.9 *p <* 0.001). HIV positive status was not associated with vaccination (OR = 1.0 *p* = 0.266). Reasons for not vaccinating included: vaccine stock-outs where ANCs depleted available stock of vaccines and/or were awaiting delivery of vaccines (54.6%, 6949/12723), refusal/indecision (25.8%, 3285), and current illness that contraindicated vaccination (19.6%, 2489).

**Conclusion:**

Antenatal vaccination uptake was likely improved by the increased vaccine supply and vaccine education offered during our campaign.

## Introduction

Among those in groups at increased risk for influenza virus infection in South Africa during 2013–2015, the estimated rates of severe influenza-associated illness were highest in pregnant women (930 per 100,000 population) [1], confirming global research showing that pregnant women are at increased risk of severe complications and hospitalisation following influenza [2–4]. Children aged less than 6 months are also at increased risk of hospitalisation and mortality from influenza-related illnesses [5, 6], yet there is no vaccine licensed for use in this age group. Maternal influenza vaccination can prevent influenza in pregnant women, as well as their young infants during the first 3–6 months of life [7–9].

In South Africa, a study to describe the national burden of influenza-associated mortality between 1999 to 2009 showed that pregnant women have an increased risk of mortality associated with seasonal as well as pandemic A(H1N1)pdm09 influenza, compared with non-pregnant women, estimating 123 all-cause seasonal influenza-associated deaths annually, and in 2009 alone, 181 pandemic A(H1N1)pdm09 influenza-associated deaths among pregnant women [4]. Influenza morbidity and mortality among pregnant women could be prevented by investing in a maternal influenza immunization strategy. Additional benefits to investing in maternal immunization include a strengthened antenatal care system and improved country pandemic preparedness [10].

Since 2010, the Department of Health (DoH) in South Africa procured approximately 1 million doses of Southern Hemisphere trivalent inactivated influenza vaccines annually to distribute among public health facilities with the aim of targeting risk groups, including pregnant women, for vaccination [11]. In 2012, the World Health Organization (WHO) recommended that pregnant women have the highest priority for influenza vaccination [12], promoting influenza vaccination as an essential component of antenatal care. In 2016, in agreement with the WHO’s recommendation and based on estimated morbidity and mortality averted, as well as estimated associated costs, South Africa’s National Advisory Group on Immunization (NAGI) prioritised pregnant women and persons with HIV infection for influenza vaccination [13]. Despite this, the national coverage for annual influenza vaccination of pregnant women since 2011 has been < 16% [11].

In South Africa, the typical influenza season occurs in the Southern hemisphere winter, starting around the first week of June and lasting between 11 to 25 weeks [14–17]. During 2015–2018, the annual routine DoH influenza vaccination programme among pregnant women was augmented at two sites in South Africa in order to increase vaccine coverage prior to a study on maternal influenza vaccine effectiveness. We describe influenza vaccine coverage among pregnant women at these sites and reasons for not receiving the vaccine, and report on a post-introduction evaluation of the programme in pregnant women. We also evaluated the timeliness of the influenza vaccination campaign each year in relation to the timing of the influenza season.

## Methods

From 2015 through 2018, the National Institute for Communicable Diseases (NICD), a division of the National Health Laboratory Services (NHLS) in South Africa, collaborated with the DoH to augment the annual routine influenza vaccination programme targeting pregnant women at selected sites. The aims were to increase influenza vaccination coverage and, as part of a larger study, aiming to measure influenza vaccine effectiveness in this population. Here we present characteristics of the annual influenza vaccination campaigns. Antenatal clinics (ANCs) were selected to participate in the campaign based on proximity to three hospitals participating in a larger study to determine maternal influenza vaccine effectiveness among infants, namely Rahima Moosa Mother and Child Hospital in Johannesburg, Gauteng Province; Red Cross War Memorial Children’s Hospital and Mitchell’s Plain District Hospital in Cape Town, Western Cape (WC) Province. In total, from the 60 ANCs serving the catchment areas of these hospitals, 25 were selected in 2015, 27 in 2016, 27 in 2017, and 26 in 2018, with the goal of increasing influenza vaccine coverage to > 50% among pregnant women giving birth at these facilities.

The annual routine influenza vaccination programme was augmented through providing additional resources as described below. Prior to the start of the influenza vaccine campaign, healthcare workers at the selected ANCs received on-site training regarding influenza, the influenza vaccine, and how to vaccinate pregnant women, including the importance of documenting vaccine administration. Additional to the allocated DoH vaccine stock, study doses of influenza vaccine procured for the exclusive vaccination of pregnant women were delivered to the selected ANCs. The vaccines procured by the study were the same vaccines used by the DoH by year as follows: 2015: Vaxigrip® IIV3 (Sanofi Pasteur, Lyon, France) and Fluvac (bioCSL, Australia); 2016: Vaxigrip® IIV3 (Sanofi Pasteur, Lyon, France); 2017: Influvac® (Abbott, Auckland, New Zealand); 2018: Influvac® (Abbott, Auckland, New Zealand). Study-employed research administrators delivered short daily health talks and provided information regarding influenza vaccination to groups of pregnant women, completed vaccine registers, and monitored vaccine supply and usage at the selected ANCs. If pregnant women refused vaccination on initial visits, they were encouraged to receive the influenza vaccine on subsequent visits during the campaign period. Between 2015 and 2018, the annual influenza vaccination programme aimed to deliver majority of influenza vaccines prior to the start of the influenza season, but actual timing of vaccination was determined by the availability and delivery of influenza vaccines to the selected sites.

### Data collection

Vaccine registers were used by DoH to document information on all pregnant women attending the ANC during the influenza vaccine campaign. Vaccine register data included patient identifiers, maternal age, HIV status, gestational age at the time of visit, vaccination status, and reason for not accepting the vaccine, if applicable. At participating ANCs, these registers were completed by the study-employed research administrators, and then captured electronically using REDCap (Research Electronic Data Capture) electronic data capture tools hosted by the University of Witwatersrand [18, 19]. The number of ANC visits per pregnant woman during the influenza vaccine campaign was also recorded for 2016–2018.

### Data analysis

We described the characteristics of pregnant women attending antenatal clinics using multivariable logistic regression, as well as reasons for not being vaccinated using univariate logistic regression. Covariates describing characteristics of pregnant women with *p*-values < 0.2 in univariate analysis were included in the multivariable model, and statistical significance was considered for *p* < 0.05. The statistical analyses were performed with Stata software, version 14.0 (StataCorp, College Station, Texas, USA). We estimated the number of influenza vaccines supplied from the DoH by subtracting the number of study doses administered from the total women vaccinated at the selected ANCs.

### Influenza surveillance data

The influenza virus data and proportions positive were obtained from the Viral Watch programme, an influenza surveillance programme coordinated by the NICD, and used to describe the timing of the 2015–2018 influenza seasons in South Africa [20]. The influenza season was considered to have started when influenza proportion positive rose above the calculated threshold, determined by the Moving Epidemic Method (a sequential analysis using the R Language) [14–17]. Dates of vaccination collected from women vaccinated at the selected ANCs between 2015 and 2018, were compared to the timing of the start of each year’s influenza season to evaluate the timeliness of each year’s vaccination campaign.

### Vaccine campaign timing and coverage

The campaign’s start date was defined as the date of the first administration of influenza vaccine in the ANCs and the end date was when the last influenza vaccine was used in the ANCs. Vaccine coverage was calculated by dividing the number of pregnant women vaccinated by the number of those that were screened at the selected ANCs within the campaign period.

### Influenza vaccine post introduction evaluation (IPIE)

IPIE tools are useful for delineating operational strengths and weaknesses of seasonal influenza vaccination programmes [21]. An influenza-focused questionnaire and IPIE tool was developed from the WHO New Vaccine PIE tool [22] to provide a systematic method for evaluating the impact of introducing the influenza vaccine on the existing immunization system. The IPIE tool targeted the following key areas: 1) Pre-introduction planning, 2) Vaccine storage and wastage, 3) Logistics of vaccine administration and data collection for coverage calculations, 4) Adverse events following immunization, 5) Training and knowledge of healthcare workers, and 6) Community and provider receptiveness. The IPIE tool and questionnaire were utilised between 6 and 10 July 2015 at the 7 WC Province ANCs that participated in the 2015 augmented annual influenza campaign, namely Hanover Park, False Bay, Retreat, Mitchell’s Plain, Mowbray, Vanguard, and Gugulethu. A team consisting of 1 field epidemiologist and 1 research administrator phoned the participating ANCs and organised meetings where interviews with the ANC managers and/ or pharmacists were conducted.

### Ethics

The augmented influenza vaccination campaign among pregnant women was part of the study of antenatal influenza vaccine effectiveness approved by the University of Witwatersrand Human Research Ethics Committee (certificate M140826) and the Faculty of Health Sciences, University of Cape Town Human Research Ethics Committee (reference 835/ 2014), and all methods were performed in accordance with the relevant guidelines and regulations. The National Health Research Committee and the WC Provincial Health Research Committee also approved the protocol. Informed consent was not required to utilise data from vaccine registers, as these were collected as part of routine healthcare practice in collaboration with the DoH. Verbal informed consent was obtained from staff participating in the IPIE campaign evaluation.

## Results

In total, from 2015 through 2018, the DoH supplied 18.5% (9125/49355) of the vaccine doses administered at the selected ANCs. Among the 62,979 pregnant women who received care and were screened at the selected ANCs during the influenza vaccination campaigns, we obtained vaccination status for 62,682 (99.5%), of which 49,355 (78.7%) were vaccinated (Table 1). Percent vaccinated was 69.7 (10,473/15017) in 2015, 86.3 (11,212/13000) in 2016, 80.2 (14,066/17545) in 2017, and 79.5 (13,604/17120) in 2018. Among 49,355 vaccinated women, 46,129 (93.5%) were vaccinated on their first antenatal visit during the influenza vaccination campaign period, 2897 (5.9%) on their second visit, and < 1% each on their third (*n* = 295), fourth (*n =* 31), or fifth (*n =* 3) visits.

**Table 1:** Characteristics of vaccinated and unvaccinated pregnant women screened in Gauteng and Western Cape, 2015-2018

Characteristics	Total n/N (%) or Median [IQR]	Vaccinated n/N (%) or Median [IQR]	Unvaccinated Total n/N (%) or Median [IQR]
*Number screened:*			
Gauteng	27,068/62979 (43.0)	18,532/49355 (37.6)	8339/13327 (62.6)
Western Cape	35,911/62979 (57.0)	30,823/49355 (62.5)	4988/13327 (37.4)
Age (years)	27 [23–32]	27 [23–31]	27 [23–32]
*Age group (years):*			
< 18	2047/62877 (3.3)	1667/49297 (3.4)	370/13292 (2.8)
18-24	20,469/62877 (32.6)	16,237/49297 (32.9)	4150/13292 (31.2)
25-29	18,295/62877 (29.1)	14,243/49297 (28.9)	3974/13292 (29.9)
30-34	13,863/62877 (22.0)	10,828/49297 (22.0)	2955/13292 (22.2)
35+	8203/62877 (13.0)	6322/49297 (12.8)	1843/13292 (13.9)
*HIV status:*			
Infected	11,287/61298 (18.4)	8861/48279 (18.4)	2390/12844 (18.6)
Gestational age at 1st screening during campaign (weeks)	28 [20–34]	27 [20–33]	28 [21–34]
*Trimester distribution at 1st screening during campaign (weeks)*			
Trimester 1: < 13	4408/52477 (8.4)	35,414/41377 (8.6)	863/11040 (7.8)
Trimester 2: 14-27	21,758/52477 (41.5)	17,520/41377 (42.3)	4210/11040 (38.1)
Trimester 3: 28-42	26,311/52477 (50.1)	20,316/41377 (49.1)	5967/11040 (54.1)

*Abbreviations: *n* numerator, *N* denominator, *%* column percentage, *IQR* interquartile range

We obtained data on gestational age at vaccination for 81.2% (40,058/49355) of vaccinated pregnant women; 8.4% (3346/40058) were vaccinated in their first trimester of pregnancy (< 13 weeks gestation), 41.0% (16430) in their second (14–27 weeks gestation), and 50.6% (20282) in their third (28 –42 weeks gestation). Both the median gestational age at first ANC visit during the campaign for all screened pregnant women, and the median gestational age at vaccination, were 28 weeks (Interquartile range (IQR) 20–34 weeks). Among those vaccinated in the third trimester, 43.0% (8717/20282) were vaccinated between 28 and 32 weeks of gestation, 37.4% (7584) between 33 and 37 weeks of gestation, and 19.6% (3981) between 38 and 42 weeks of gestation.

On multivariable analysis (Table 2), compared to unvaccinated pregnant women, vaccinated women were more likely to be from the WC (OR 3.7, 95% CI 3.5–3.8), less likely to be aged 25–29 (OR 0.9, 95% CI 0.9–1.0) or ≥ 35 years (OR 0.9, 95% CI 0.8–0.9) compared to 18–24 years, and more likely to be in the first (OR 1.3, 95% CI 1.2–1.5) or second (OR 1.5, 95% CI 0.8–0.9) trimester compared to the third trimester.

**Table 2:** Multivariable analysis comparing vaccinated and unvaccinated pregnant women screened in Gauteng and Western Cape, 2015-2018

Characteristics	OR (95% CI)	*P-*value	Adjusted OR (95% CI)	*P-*value
*Number screened:*				
Gauteng	Reference	Reference	Reference	Reference
Western Cape	2.8 (2.7–2.9)	< 0.001	3.7 (3.5–3.8)	< 0.001
Age (years)	–	0.015	–	–
*Age group (years):*				
< 18	1.2 (1.0–1.3)	0.019	1.0 (0.9–1.2)	0.987
18-24	Reference	Reference	Reference	Reference
25-29	0.9 (0.9–1.0)	< 0.001	0.9 (0.9–1.0)	0.053
30-34	0.9 (0.9–1.0)	0.015	1.0 (0.9–1.0)	0.554
35+	0.9 (0.8–0.9)	< 0.001	0.9 (0.8–0.9)	< 0.001
*HIV status:*				
Infected	1.0 (0.9–1.0)	0.509	–	–
Gestational age at 1st screening during campaign (weeks)	–	< 0.001	–	–
*Trimester distribution at 1st screening during campaign (weeks)*				
Trimester 1: < 13	1.2 (1.1–1.3)	< 0.001	1.3 (1.2–1.5)	< 0.001
Trimester 2: 14-27	1.2 (1.12–1.3)	< 0.001	1.5 (1.4–1.5)	< 0.001
Trimester 3: 28-42	Reference	Reference	Reference	Reference

*Abbreviations: *n* numerator, *N* denominator, *%* column percentage, *IQR* interquartile range, *CI* confidence interval, *OR* odds ratio

Among the 13,327 pregnant women not vaccinated, 12,723 (95.5%) reported the reason for non-vaccination, including vaccine stock-outs, that was not a participant decision but where ANCs depleted available stock of vaccines and/ or were awaiting delivery of vaccines (54.8%, 6949/12723), refusal/ indecision (25.8%, 3285), and current illness that contraindicated vaccination (19.6%, 2489). On univariate analysis (Table 3) comparing reasons for non-vaccination, vaccine stock-outs (OR 1.3, 95% CI 1.2–1.5) and current illness (OR 1.5, 95% CI 1.4–1.5) were more likely to be a reason for non-vaccination compared to refusal/ indecision in Gauteng compared to WC.

**Table 3:** Univariate logistic regression analysis comparing reasons for non-vaccination among unvaccinated pregnant women screened in Gauteng and Western Cape, 2015-2018

Reasons for not receiving the influenza vaccine:	Gauteng (n/N, %)	WC (n/N, %)	OR (95% CI)	***P-***value
Vaccine stock-outs	4398/7793 (56.4)	2551/4930 (51.7)	1.3 (1.2–1.4)	< 0.001
Refusal/ indecision	1860/7793 (23.9)	1425/4930 (28.9)	–	–
Current illness	1535/7793 (19.7)	954/4930 (19.4)	1.2 (1.1–1.4)	< 0.001

*Abbreviations: *n* numerator, *N* denominator, *%* column percentage, *OR* odds ratio, *CI* confidence interval, *WC* Western Cape

Seven nursing managers from the 7 ANCs in WC were interviewed for the IPIE, and although they mostly reported that influenza vaccination during pregnancy had minimal negative impact on the provision of routine services during the augmented campaign, they indicated that services could have benefited from the timely training of antenatal staff by the DoH, additional educational resources, and an increased supply of vaccines (Table 4).

**Table 4:** Summary of the key findings using the IPIE tool at 7 ANCs in Western Cape, 2015

Key Areas	Key findings
Planning and training	• None of the sites reported receiving training from the DoH on the vaccination campaign in the current year • Influenza vaccine coverage and waste calculation could not be determined at the site level • Several sites complained about the documentation process, specifically citing placing stickers and verifying vaccine receipt in the record books as “too labour intensive”
Vaccine management	• Cold chain management was uniform across sites • Vaccine shortage was an issue at 2 (29%) sites • Vaccine wastage reported at 2 (29%) sites due to failure to refrigerate
Adverse events	• One AEFI* of localised injection site rash was identified. This was reported to a medical officer only and not documented using the national AEFI reporting mechanism (through the pharmacy)
Impression and acceptance	• Overall, the nurse managers interviewed were pleased with their involvement in the campaign • Of 7 sites surveyed, 6 (85%) felt implementation of the campaign was a smooth process with minor challenges and did not affect their ability to provide routine antenatal services
Recommendations	• Study RAs should vaccinate patients to decrease the burden on the clinic staff • Provide more influenza educational materials (checklists, presentations geared toward a lay audience) for use during the season

*Abbreviations: *DoH* Department of Health, *AEFI* Adverse event following immunization, *RA* research assistant

**Fig. 1:**
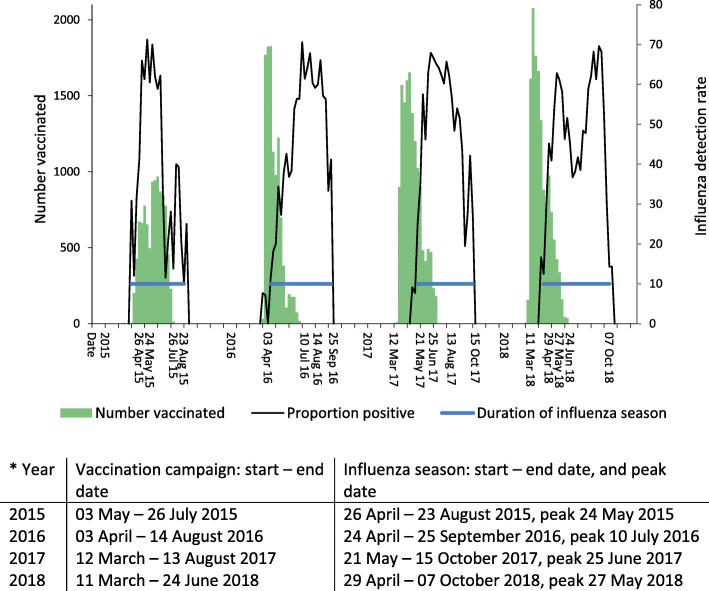
Number of pregnant women vaccinated compared with influenza proportion positives by epidemiologic week, and duration of the influenza season indicated by the blue line, in Gauteng and Western Cape, 2015–2018.*

Amongst women with data available on date of vaccination (48,068/49355, 97.4%), half of vaccines (51.9%, 24,944/48068) were administered prior to the start of the influenza season and 18.9% (9072/48068) were administered after the annual seasonal influenza peak. However, in 2015, only 2.1% (210/ 9931) of influenza vaccines were administered prior to the start and 7183 (72.3%) after the peak of the influenza season (Fig. 1).

## Discussion

Between 2015 and 2018, we successfully increased influenza vaccine coverage among pregnant women at participating ANCs. By increasing the number of vaccine doses available and providing clinic staff with basic training on the benefits of influenza vaccination to pregnant women, we were able to vaccinate most (78.7%) pregnant women in study clinics. In spite of this, around half (54.8%) of screened pregnant women that did not receive the influenza vaccine reported vaccine unavailability/ stock-outs as a reason for non-vaccination suggesting a health system failure, that if addressed might have increased coverage even further.

The high vaccine coverage achieved and the fact that the majority of vaccinated pregnant women (93.5%) were vaccinated on their first antenatal visit during the vaccination campaign, suggests that influenza vaccine uptake among pregnant women at ANCs was well accepted. Especially where additional resources such as free and increased vaccine supply, focused training of healthcare workers and improved maternal vaccine education were supplied, a high vaccine coverage was achieved. These findings are similar to those from Alessandrini et al. [23] where the provision of free influenza vaccine increased vaccine coverage significantly. In other studies, programmes focussing on maternal knowledge of vaccines have also improved the timing, completeness, and coverage of vaccination [24]. Previous research has shown that by improving healthcare worker’s knowledge of influenza vaccines, their willingness to recommend vaccination to pregnant women will increase vaccine uptake, and that their advice is a key determinant of influenza vaccination uptake in pregnant women [25–28].

There are several challenges affecting optimal timing of influenza vaccination, including vaccine manufacturing issues. In 2015, due to a change in influenza strains included in the vaccine, there was a delay in manufacturing and subsequently receiving the Southern Hemisphere influenza vaccines [29], resulting in the vaccination campaign only being able to start in May. Unfortunately, 2015 also saw an early onset to the influenza season [16], starting 26 April, 1 week before the vaccination campaign began, and peaking the week of 24 May. This resulted in the majority (72.3%) of influenza vaccines being administered to pregnant women after the seasonal influenza peak, which would likely reduce the vaccine’s impact to protect mother and child. In contrast, during 2016–2018 the majority of influenza vaccines were administered before the onset of the annual influenza season, with less than 5% being administered after the seasonal peak.

When considering timing relative to gestational age at vaccination, research has shown that increased antibody levels in cord blood are significantly associated with pregnant women vaccinated in 2nd and 3rd trimesters compared to those vaccinated in the 1st trimester, although titres are significantly lower if the pregnant woman was vaccinated less than 4 weeks before birth, concluding that regardless of trimester, influenza vaccines should be administered during pregnancy as soon as they become available [30]. The newer WHO ANC guidelines recommend 8 rather than 4 ANC visits in pregnancy [31]. Although the majority of pregnant women were vaccinated on their first antenatal visit during the campaign, this proposed increase in visits may provide additional opportunities for vaccination and timing closer to the start of influenza season. Of those vaccinated pregnant women with gestational ages available, the majority were vaccinated in their 3rd trimesters (50.6%), with a further breakdown showing that most 3rd trimester vaccinations (43%) occurred before 33 weeks gestational age.

There were several limitations to our study. First, in ANCs where screening occurred after the pregnant woman’s examination, pregnant women refusing vaccination and wanting to avoid screening may have exited the clinic unnoticed, leading to overestimating vaccination coverage. The impact of this may have been reduced if these women were screened on subsequent visits, as South African government indicators for maternal health in 2016 reported that 73.2% pregnant women from urban areas attend four or more antenatal visits [32]. Second, different methods of assessing gestational age at ANC clinics may have impacted the estimation of gestational age at first screening. A third limitation is that the IPIE was limited to the WC Province only, targeting 7 out of a possible 27 participating ANC clinics. Finally, there may have been some social desirability bias present where non-vaccinated pregnant women were hesitant to choose their true reason for vaccine refusal, from a list of possibilities, in front of staff.

## Conclusion

Previous research has shown that antenatal influenza vaccination campaigns in South Africa can reduce the impact of influenza and be cost-effective [33]. This study shows that maternal influenza vaccination was well-accepted among pregnant women and healthcare workers, and that improving vaccine supply and provider education could substantially improve the uptake and overall coverage of the influenza vaccine among pregnant women.

## Data Availability

The datasets used and/or analysed during the current study are available from the corresponding author on reasonable request.

## Abbreviations

AEFI: Adverse event following immunization
ANC: Antenatal Clinic
CI: Confidence interval
DoH: Department of Health
IPIE: Influenza vaccine Post Introduction Evaluation
IQR: Interquartile range
n: Numerator
N: Denominator
NAGI: National Advisory Group on Immunization
NHLS: National Health Laboratory Service
NICD: National Institute for Communicable Diseases
OR: Odds ratio
RA: Research assistant
REDCap: Research Electronic Data Capture
WC: Western Cape
WHO: World Health Organization
%: Column percentage

## References

[1] Tempia S, Walaza S, Moyes J (2020). Influenza disease burden among potential target risk groups for immunization in South Africa, 2013-2015. Vaccine..

[2] Jamieson DJ, Honein MA, Rasmussen SA (2009). H1N1 2009 influenza virus infection during pregnancy in the USA. Lancet..

[3] Louie JK, Acosta M, Jamieson DJ, Honein MA (2010). Severe 2009 H1N1 influenza in pregnant and postpartum women in California. N Engl J Med.

[4] Tempia S, Walaza S, Cohen AL (2015). Mortality associated with seasonal and pandemic influenza among pregnant and nonpregnant women of childbearing age in a high-HIV-prevalence setting-South Africa, 1999-2009. Clin Infect Dis.

[5] Bhat N, Wright JG, Broder KR (2005). Influenza-associated deaths among children in the United States, 2003-2004. N Engl J Med.

[6] Neuzil KM, Mellen BG, Wright PF, Mitchel EF, Griffin MR (2000). The effect of influenza on hospitalizations, outpatient visits, and courses of antibiotics in children. N Engl J Med.

[7] Madhi SA, Cutland CL, Kuwanda L (2014). Influenza vaccination of pregnant women and protection of their infants. N Engl J Med.

[8] Tapia MD, Sow SO, Tamboura B (2016). Maternal immunisation with trivalent inactivated influenza vaccine for prevention of influenza in infants in Mali: a prospective, active-controlled, observer-blind, randomised phase 4 trial. Lancet Infect Dis.

[9] Zaman K, Roy E, Arifeen SE (2008). Effectiveness of maternal influenza immunization in mothers and infants. N Engl J Med.

[10] Ortiz JR, Neuzil KM (2017). Influenza immunization of pregnant women in resource-constrained countries: an update for funding and implementation decisions. Curr Opin Infect Dis.

[11] Ramkrishna W, Moonasar D, Tempia S, McMorrow ML, Walaza S, Cohen C (2019). Policy implementation to improve seasonal influenza vaccination of target groups in South Africa, 2011-2018 [Unpublished].

[12] Meeting of the Strategic Advisory Group of Experts on immunization (2012). April 2012--conclusions and recommendations. Wkly Epidemiol Rec.

[13] McMorrow ML, Tempia S, Walaza S (2019). Prioritization of risk groups for influenza vaccination in resource limited settings - a case study from South Africa. Vaccine..

[14] Moyes J, Walaza S, Chikosha S, et al. Public Health Surveillance Bulletin: Epidemiology of respiratory pathogens from influenza-like illness and pneumonia surveillance programmes, South Africa, 2018. National Institute for communicable diseases. Public Health Surveillance Bulletin Web site. https://www.nicd.ac.za/wp-content/uploads/2019/05/NICD-Bulletin-Vol17-Iss1-April-2019-FINAL.pdf Accessed 12 December 2020.

[15] Walaza S, Buys A, Cohen C, et al. Public Health Surveillance Bulletin: Epidemiology of respiratory pathogens from influenza-like illness and pneumonia surveillance programmes, South Africa, 2017. . National Institute for communicable diseases. Communicable Diseases Surveillance Bulletin Web site https://wwwnicdacza/wp-content/uploads/2018/10/NICD-Bulletin-Vol16-Iss1-April-2018-Finalpdf Accessed 12 December 2020.

[16] Walaza S, Cohen C, Treurnicht F, et al. Public Health Surveillance Bulletin: Burden of respiratory pathogens from influenza like illness and pneumonia surveillance programmes, South Africa, 2015. . National Institute for communicable diseases. Communicable Diseases Surveillance Bulletin Web site https://wwwnicdacza/assets/files/CommDisBull%2014(1)-Mar2016(1)pdf Accessed 24 November 2020.

[17] Walaza S, Cohen C, Treurnicht F, et al. Public Health Surveillance Bulletin: Epidemiology of respiratory pathogens from influenza-like illness and pneumonia surveillance programmes, South Africa, 2016. . National Institute for communicable diseases. Public Health Surveillance Bulletin Web site https://wwwnicdacza/wp-content/uploads/2019/05/NICD-Bulletin-Vol17-Iss1-April-2019-FINALpdf Accessed 24 November 2020.

[18] Harris PA, Taylor R, Minor BL (2019). The REDCap consortium: Building an international community of software platform partners. J Biomed Inform.

[19] Harris PA, Taylor R, Thielke R, Payne J, Gonzalez N, Conde JG (2009). Research electronic data capture (REDCap)--a metadata-driven methodology and workflow process for providing translational research informatics support. J Biomed Inform.

[20] McAnerney JM, Cohen C, Moyes J (2012). Twenty-five years of outpatient influenza surveillance in South Africa, 1984-2008. J Infect Dis.

[21] World Health O (2010). New vaccine post-introduction evaluation (PIE) tool.

[22] World Health O. IPIE manual and tool. World Health Organization https://www.who.int/immunization/research/development/ipie_influenza_post_introduction_evaluation/en/. Accessed 22 January 2021.

[23] Alessandrini V, Anselem O, Girault A (2019). Does the availability of influenza vaccine at prenatal care visits and of immediate vaccination improve vaccination coverage of pregnant women?. PLoS One.

[24] Hu Y, Chen Y, Wang Y, Song Q, Li Q (2017). Prenatal vaccination education intervention improves both the mothers' knowledge and children's vaccination coverage: evidence from randomized controlled trial from eastern China. Hum Vaccin Immunother.

[25] Mak DB, Regan AK, Joyce S, Gibbs R, Effler PV (2015). Antenatal care provider's advice is the key determinant of influenza vaccination uptake in pregnant women. Aust N Z J Obstet Gynaecol.

[26] Prospero E, Galmozzi S, Paris V (2019). Factors influencing refusing of flu vaccination among pregnant women in Italy: healthcare workers' role. Influenza Other Respir Viruses.

[27] Yuet Sheung Yuen C, Yee Tak Fong D, Lai Yin Lee I, Chu S, Sau-mei Siu E, Tarrant M (2013). Prevalence and predictors of maternal seasonal influenza vaccination in Hong Kong. Vaccine..

[28] Duque J, Gaga S, Clark D (2017). Knowledge, attitudes and practices of south African healthcare workers regarding the prevention and treatment of influenza among HIV-infected individuals. PLoS One.

[29] NIOH. Topcial Issues 2015: Influenza vaccine availability for the 2015 influenza season. National Institute for Occupational Health. https://www.nioh.ac.za/influenza-vaccine-availability-for-the-2015-influenza-season/. Accessed 22 January 2021.

[30] Zhong Z, Haltalli M, Holder B (2019). The impact of timing of maternal influenza immunization on infant antibody levels at birth. Clin Exp Immunol.

[31] WHO. WHO recommendations on antenatal care for a positive pregnancy experience. World Health Organization. Guideline Web site. https://www.who.int/publications/i/item/9789241549912. Published 2017. Accessed March 08, 2021. 28079998

[32] StatsSA. Maternal Health Indicators. stats sa. http://www.statssa.gov.za/?p=13100. Published 2020. Accessed 22 January 2021.

[33] Biggerstaff M, Cohen C, Reed C (2019). A cost-effectiveness analysis of antenatal influenza vaccination among HIV-infected and HIV-uninfected pregnant women in South Africa. Vaccine..

